# Novel Formulations for Antimicrobial Peptides

**DOI:** 10.3390/ijms151018040

**Published:** 2014-10-09

**Authors:** Ana Maria Carmona-Ribeiro, Letícia Dias de Melo Carrasco

**Affiliations:** Biocolloids Laboratory, Instituto de Química, Universidade de São Paulo, Av. Lineu Prestes 748, 05508-000 São Paulo, SP, Brazil; E-Mail: lemelodias@usp.br

**Keywords:** bilayer disks or fragments, liposomes, nanoparticles, biocompatible polymers, peptide covalent modification, biopolymers

## Abstract

Peptides in general hold much promise as a major ingredient in novel supramolecular assemblies. They may become essential in vaccine design, antimicrobial chemotherapy, cancer immunotherapy, food preservation, organs transplants, design of novel materials for dentistry, formulations against diabetes and other important strategical applications. This review discusses how novel formulations may improve the therapeutic index of antimicrobial peptides by protecting their activity and improving their bioavailability. The diversity of novel formulations using lipids, liposomes, nanoparticles, polymers, micelles, *etc.*, within the limits of nanotechnology may also provide novel applications going beyond antimicrobial chemotherapy.

## 1. Structure and Function of Antimicrobial Peptides

**Figure 1:**
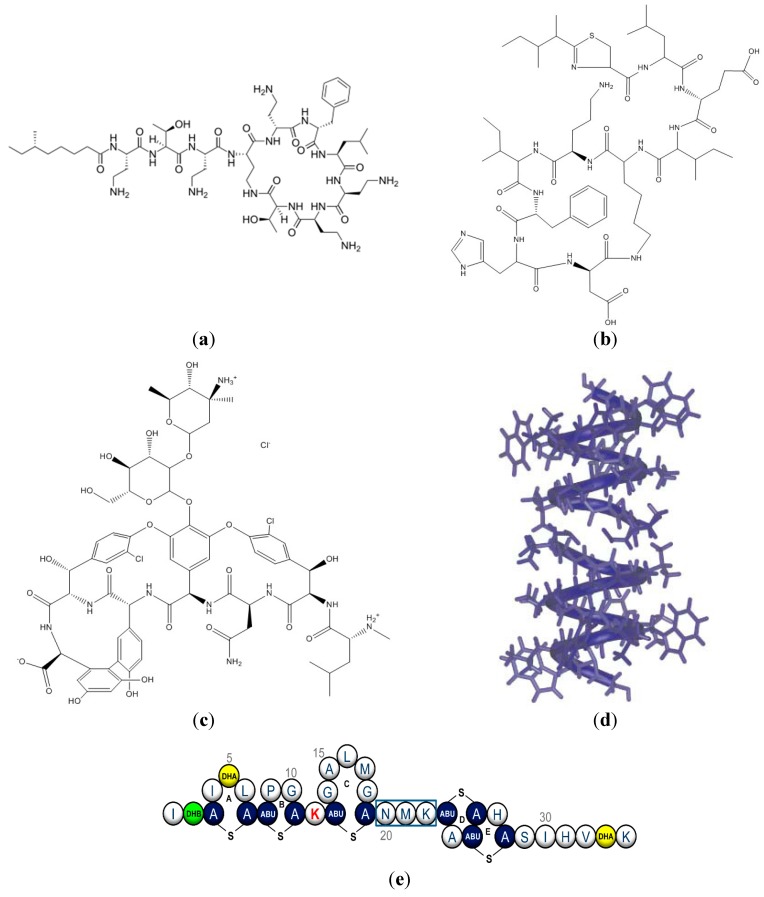
(**a**) Polymyxin B; (**b**) Bacitracin; (**c**) Vancomycin; (**d**) Gramicidin A as a peptide dimer traversing the membrane and anchoring to the membrane interface by its four Trp residues. Adapted from [19] with permission from Elsevier, Copyright 2005; (**e**) Nisin with its unusual aminoacids such as dehydroalanine (DHA), dehydrobutyrine (DHB) and several intramolecular thio-ether bridges. Reprinted from [20].

The growing challenge of microbial resistance emphasizes the importance of novel antibiotics or new assemblies for old ones. With emerging infectious diseases as a major public health problem, the research on antimicrobial peptides (AMPs) is timely and much work has been devoted to AMPs over the last three decades with excellent reviews available [1,2,3,4,5,6,7,8,9,10,11,12,13]. Antimicrobial peptides are either non-ribosomally synthesized peptides (NRAMPs) [14] or ribosomally synthesized peptides (RAMPs) [15]. NRAMP synthesis catalyzed by peptide synthetases takes place in the cytosol of bacteria and fungi [14] whereas RAMP synthesis occurs in the ribosomes of the eukaryotic cells [15]. Polymyxin B [16], bacitracin [17], vancomycin [18] and gramicidin A [19] are examples of NRAMPs whereas nisin [20] is a gene-encoded RAMP. Figure 1 shows some structural features of these peptides.

**Figure 2:**
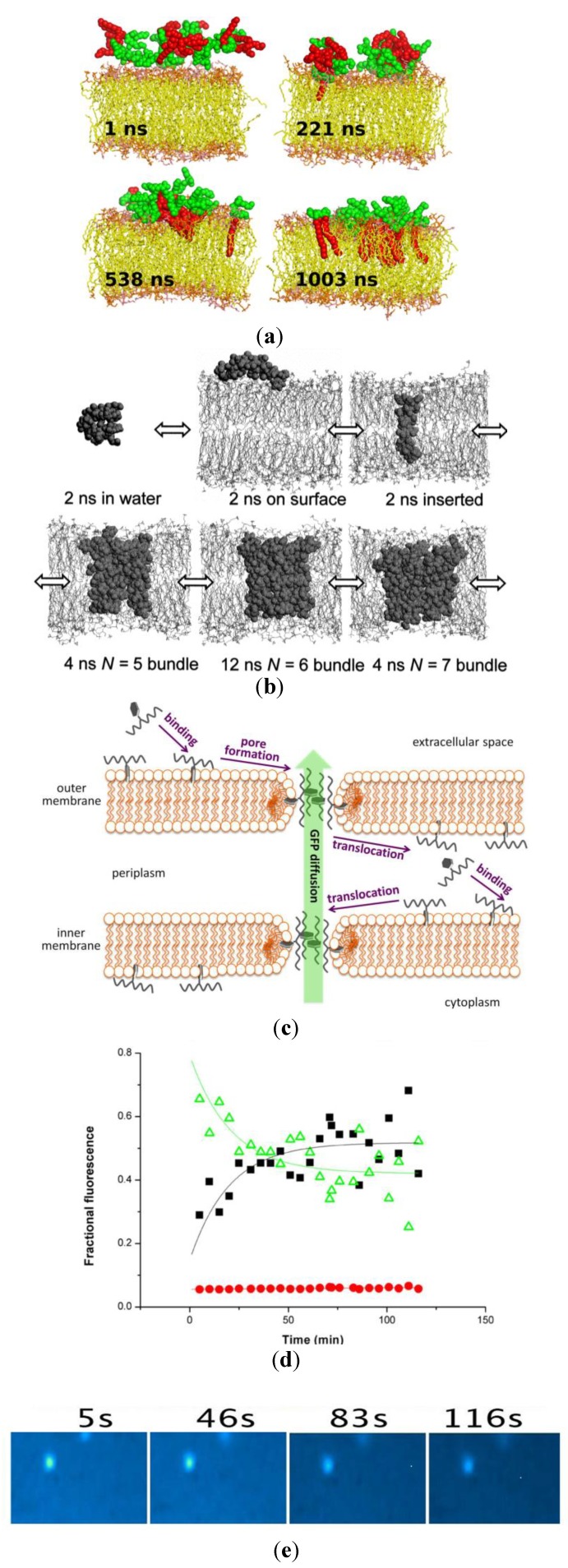
(**a**) Molecular dynamics simulation of the C16-KGGK lipopeptide interacting with a lipid bilayer. Reprinted from [30] with permission from American Chemical Society, Copyright 2013; (**b**) Alamethicin and its multichannel bundles. Adapted from [31] with permission from Elsevier, Copyright 1999; (**c**) A derivative of mellitin (mellitin K14) interacting with the *Escherichia coli* double membranes [32]. Reprinted from [32] by permission Macmillan Publishers Ltd., London, UK, Copyright 2013; (**d**) The polarity-sensitive fluorescent probe AlexaFluor-430 covalently bound at the K14 residue 26 yields the kinetics of fractional fluorescence from: free melittin (black squares); lipidic pore melittin (red circles); and from the *E. coli* bacterial cytoplasm and within the cell wall (green triangles). Adapted from [32] by permission from Macmillan Publishers Ltd., London, UK, Copyright 2013; (**e**) The decrease in fluorescence in a single *E. coli* cell due to leakage of green fluorescent protein GFP through the mellitin pores in the membrane. Adapted from [32] by permission from Macmillan Publishers Ltd., London, UK, Copyright 2013.

Several species, from prokaryotes to humans, synthesize AMPs since they act as a host’s natural defense against the daily exposure to millions of pathogens. All higher eukaryotes produce molecules for defense against microbes [1,21,22]. RAMPs may also possess antiviral, antiparasitic, antineoplastic and immunomodulatory activity. Among the several substances produced are the antimicrobial peptides (AMPs) [21,22]. AMPs display a variety of molecular structures [1]. There are linear peptides structured as amphipathic and hydrophobic helices, small proteins with β-sheet secondary structures, cyclic peptides and structures, peptides with unique amino acid compositions, lipopeptides, macrocyclic peptides and peptides self-assembling as bundles of α-helical rods in lipid bilayers [1]. A common secondary structure for bacteriostatic peptides is the cationic amphipathic helix [23]. However, there are also α-helical peptides that are hydrophobic or anionic displaying less selectivity towards microbes compared with mammalian cells. An example of a well-studied hydrophobic and negatively charged cytotoxic peptide is alamethicin. This helical peptide forms hexameric clusters of helices that traverse the bilayer and surround an aqueous pore [24,25]. Another peptide that is hydrophobic and forms a helical transmembrane channel is gramicidin A. Its cation-selective right-handed helix traverses the bilayer membrane as a single-stranded head-to-head dimer [24,26]. Both alamethicin and gramicidin are NRAMPs. Since these peptides exhibit little selectivity for microbial membranes, they require novel formulations or covalent modifications to become useful in antimicrobial chemotherapy. For example, gramicidin A formulated in antimicrobial cationic bilayer disks or fragments displays a substantial broadening of antimicrobial activity spectrum by selectively killing both Gram-positive and negative bacteria but shows low toxicity against the eukaryotic yeast *Saccharomyces cerevisae* [27,28]. Alternatively, chemical modifications of the gramicidin A structure also reduce its toxicity against mammalian cells while keeping its antimicrobial action [29]. Gramicidin A derivatives with the D-leucines at positions 10, 12 and 14 replaced by lysins have improved solubility in water and become cationic without altering the channel structure [29]. These derivatives achieved bacterial specificity and low toxicity against mammalian cells [29]. Figure 2 illustrates the interaction of three different peptides with the bilayer membrane illustrating the non-specific mechanism of action for cell-penetrating peptides [30,31,32].

Defensins in mammals are AMPs that are part of the innate immune system for protection against infection [33,34,35,36,37]. The inhibition of AMP activation increases wound colonization by *Staphylococcus epidermidis* in pigs [33], *Salmonella* virulence in mice correlates with a natural resistance to AMP action [34], *Shigella* infections in humans correlates with down regulation of enteric cathelicidin and β-defensin-1 expression [35] and overexpression of a human AMP gene in transgenic mice improves lung clearance of *Pseudomonas aeruginosa* [36]. Further, AMP can also recruit leukocytes to participate in cell-mediated defense [38,39]. Although much studied for their direct antimicrobial activities, AMP clinical potential might go beyond the treatment of antibiotic-resistant infections [40]. Many mammalian antimicrobial or host defense peptides stimulate the host’s immune cellular response aiding in the clearance of invading pathogens [41]. A fragment of the bacteriostatic cecropin B, despite being nonbacteriostatic accelerates murine wound repair [42].

The non-specific and destructive mechanism of action for cell-penetrating peptides show therapeutic potential against cancer and certain cationic AMP can produce tumor cell death by apoptosis via mitochondrial membranes disruption and/or preventing angiogenesis [43]. Analogs of naturally occurring frog skin host-defense peptides show selective cytotoxicity against tumor cells, and so have potential for development into anti-cancer agents [44]. Magainin-2 shows tumoricidal activity against human small cell lung cancer cell lines [45], some bladder cancer cells [46], and against a wide range of hematopoietic cell lines [47]. Some peptides secreted by frog skin with a high activity against multiresistant *Staphylococcus aureus* did not succeed as anti-infective agents due to their high hemolytic activities against human red blood cells and their rapid clearance from the circulation [48]. Thus, the therapeutic potential of frog skin peptides as anti-infective agents has not been realized so that alternative clinical applications as anti-cancer [43,44,45,46,47,48,49,50,51] or antiviral [44,49] drugs are being explored.

Nisin is a class Ia lantabiotic, a bacteriocin with several unusual amino acids due to enzyme-mediated post-translational modification [52]. These lanthionine-containing antibiotics or “lantibiotics” have the amino acids DHA and DHB formed by dehydration of serine and threonine residues [53]. Specific additional reactions between cysteine residues and some of these unsaturated amino acids lead to the formation of the characteristic lanthionine and β-methyllanthionine residues. The thio-ether bridges of these residues act as intramolecular cross-links introducing “rings” in the mature bacteriocin [54]. Nisin is safe and extensively used in the food industry for processed cheese, dairy products and canned foods [55]. It has been approved by the EU as additive E234, as well as by the World Health Organization (WHO) and the US Food and Drug Administration (FDA). As a chemotherapeutic agent, nisin has a high potency against Gram-positive pathogens (with activity at nanomolar concentrations) [56,57] and is stable and non-cytotoxic against mammals [58]. Nisin is gene-encoded, facilitating the synthesis of novel derivatives and their screening for desirable properties. Recently, site-saturation mutagenesis created a bank of producers of nisin A derivatives differing with respect to the identity of residue 12 (normally lysine; K12) and leading to the identification of derivatives with enhanced antimicrobial activity [20]. A number of these producers exhibited enhanced bioactivity as the nisin A K12A producer. Subsequent investigations with the purified antimicrobial highlighted the enhanced specific activity of this modified nisin against representative target strains from the genera Streptococcus, Bacillus, Lactococcus, Enterococcus and Staphylococcus [20]. 

Whereas class-I bacteriocins such as nisin often contain methyl-lanthionine, and other non-standard residues such as dehydroalanine, dehydrobutyrine and d-alanine, class-II consists of the non-lanthionine-containing peptide bacteriocins that are not subject to extensive post-translational modifications. Class II bacteriocins are small peptides that do not contain modified residues and display activity against foodborne pathogens such as *Listeria monocytogenes*, the deadliest bacterial source of food poisoning [52,59]. Other pathogenic bacteria inhibited by some class IIa bacteriocins include *Bacillus cereus*, *Clostridium botulinum*, *Clostridium perfringens* and *S. aureus* [60] and vancomycin-resistant enterococci [61]. These bacteriocins have also anti-cancer [62] and antiviral activity [63]. Rather complete reviews are available on bacteriocins, their classes and their structure-activity relatioships [64,65,66].

**Figure 3:**
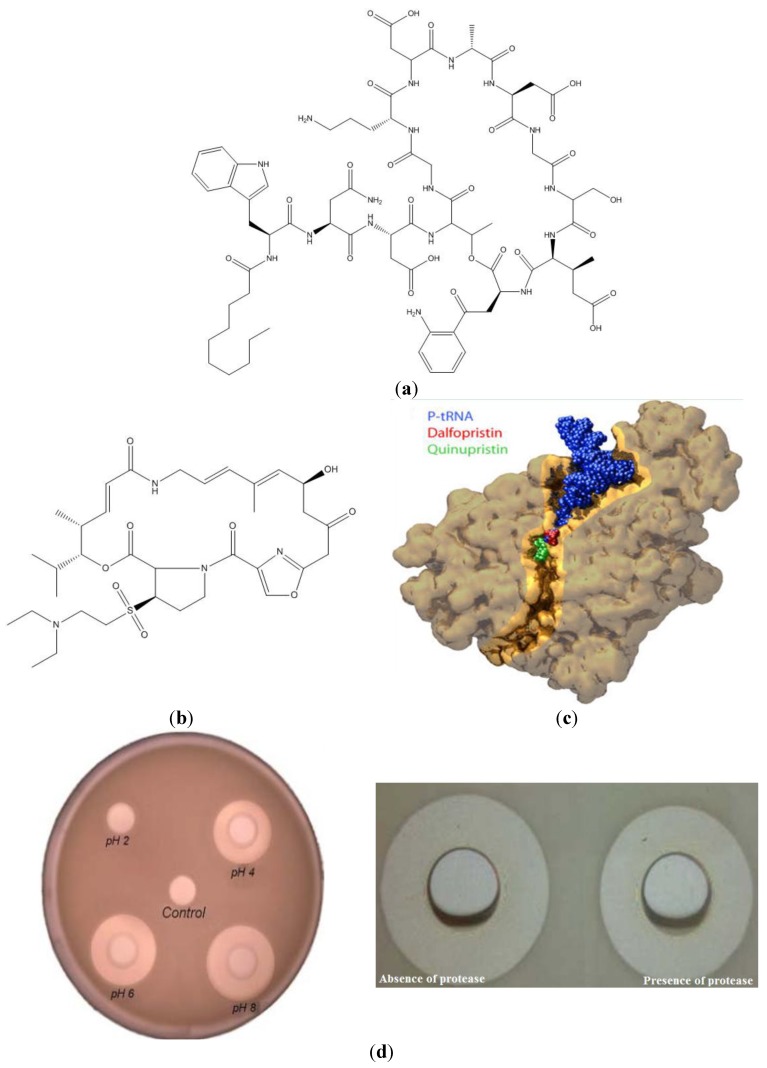
(**a**) Daptomycin (Dpt) chemical structure; (**b**) Chemical structure of dalfopristin; (**c**) Quinupristin and dalfopristin binding to the 50S ribosomal subunit in relation to the *p*-site tRNA and the ribosomal exit tunnel (highlighted in gold). Adapted from [84]; (**d**) Inhibition halos induced by *B. megaterium* CLP against a sensitive *B. cereus* strain at different pH values and in the absence or in the presence of a protease. Adapted from [97].

Among the cyclic NRAMP lipopeptides (CLP) are the lipodepsipeptides already marketed or in advanced stages of clinical trials [67]. Daptomycin (Dpt), for example, is an acidic CLP where the depsipeptide portion contains a 13 amino acid chain linked by an ester bond between the carboxyl group of l-kynurenine13 (kyn) and the hydroxyl group of l-Thr4 to form a 10 amino acid ring with a three amino acid tail [68]. The cloning and sequence analysis of the Dpt cluster and adjacent regions from *Streptomyces roseosporus* has provided a map of the organization of the genes and enzymes involved in Dpt biosynthesis. The cloned Dpt sequences ultimately have been providing a means for genetically engineering the Dpt peptide assembly for greater efficiency facilitating the generation of novel derivatives toward new antibiotics [69]. Dpt depends on calcium ions to insert deeply in the bacterial membrane and form aggregates and channels which ultimately kill the bacterium cell [70]. The chemical structure of Dpt taken from [68] is shown in Figure 3a. The binding of calcium ions does not result in major conformational changes, but does induce Dpt aggregation [69]. Dpt is approved in the US, Europe and Canada for treating infections by Gram-positive bacteria [71,72,73,74,75]. However, there are several reports of bacterial resistance against Dpt [76,77,78,79,80] with excellent reviews on Dpt resistance available [67,81] and also some reports on resistance against others lipodepsipeptides [82].

Streptogramins are CLP produced by the genus *Streptomyces* composed of macrocyclic lactone rings. Type B streptogramins (SBs) are cyclic hexadepsipeptides whereas type A (SA) are highly modified cyclopeptides with multiple conjugated double bonds [83,84]. Alone, each compound exhibits a moderate bacteriostatic activity, but in combination the synergistic interplay between SA and SB produces a bactericidal effect [85,86]. Type B streptogramins act on the 50S ribosomal subunit in a similar fashion as the macrolides and compete for the same binding site. The SBs do not affect the peptidyl transferase reaction, but inhibit elongation after a few cycles of peptide bond formation; by analogy with the macrolides, SBs bind within the tunnel and block the path of the nascent polypeptide chain. SA prevents protein biosynthesis by blocking peptide bond formation at the peptidyl transferase centre of the ribosome [87]. The 30:70 combination of dalfopristin (SA) and quinupristin (SB) approved as Synercid^®^ displays activity against Gram-positive as well as Gram-negative bacteria. The structure of the 50S ribosomal subunit from *Deinococcus radiodurans* (D50S) in complex with both dalfopristin and quinupristin was solved with 3.4 Å resolution showing unambiguously the localization of dalfopristin and quinupristin in the core region of the 50S ribosomal subunit [84] as shown in Figure 3b. Resistance of Gram-positive bacteria to the streptogramin quinupristin-dalfopristin (Q-D) involves several pathways, including drug modification, drug inactivation and drug efflux by pumps whereas resistance to Dpt has been shown to involve altered interactions with the cell membrane [88].

Polymyxins are also CLPs isolated from *Bacillus polymyxa* [89]. Their chemical structure is shown in Figure 1a. Polymyxin B is a mixture of at least four closely related components, polymyxin B1 to B4, with polymyxin B1 and B2 being the two major components [90,91]. The four components differ from each other only in the fatty acid moiety [90,91]. They are the last-line therapy against Gram-negative bacteria and clinicians worldwide are confronted by the reality of infections with Gram-negative pathogens that are resistant to all antibiotics except polymyxins [92,93]. The polymyxin molecular structure consists of the hydrophobic *N*-terminal fatty acyl chain; the positively charged side chains; the linear tripeptide segment; the hydrophobic moiety at positions 6 and 7 in the cyclic heptapeptide ring; and the heptapeptide backbone [92]. The mechanism of antimicrobial action involves the electrostatic attraction between the positive charge of the five diaminobutiric acid (Dab) residues of the polymyxin molecule and the negatively charged phosphate groups on lipid A at the outer membrane of the bacteria [94]. Modifications of lipid A phosphates with positively charged groups, such as 4-amino-4-deoxy-l-arabinose and/or phosphoethanolamine represent the most common mechanism of resistance in Gram-negative bacteria [95,96]. In the polymyxin B-lipopolysaccharide complex, the heptapeptide ring acts as a scaffold for electrostatic and hydrophobic interactions with the outer membrane of the bacteria [92,94].

The genus *Bacillus* seems to include several interesting microbial cell factories producing a variety of novel CLP products. One example is *Bacillus megaterium*, a soil megabacterium found to produce and secrete a mixture of anionic CLP. The mixture analysis by electron spray ionization (ESI) and matrix-assisted laser desorption ionization-time of flight (MALDI-TOF) mass spectrometry (MS) yields peaks at *m*/*z* 1041 and 1065 compatible with surfactins and lichenysins, respectively, whereas two other peaks *m*/*z* 1057 and 1464 detected by collision-induced dissociation (CID) unveil iturin A and fengycins A,B, respectively [97,98]. These CLPs are acidic and display a variety of lengths for the fatty acid moiety, self-assembling in aqueous solution as large, compact and negatively charged aggregates active against other *Bacillus* species upon increasing the pH [97]. They show antimicrobial and lytic activity against other *Bacillus* species such as a sensitive *B. cereus* strain as evaluated from inhibition halos and *B. cereus* lysis [97,98]. Essential features determining the antibiotic activity on susceptible *B. cereus* cells are the medium pH and the preserved cyclic moiety conferring CLP resistance to proteases [97]. The aggregates are inactive *per se* at the pH of the culture medium, which is around 6 or below. The knock out of the sensitive cells only takes place when the aggregates disassemble due to a high negative charge at pH 6 or above [97]. The pH effect on CLP activity and the CLP resistance to a protease are shown in Figure 3c.

In summary, the robust AMP activity described since the discovery of magainins, cecropins and defensins about 30 years ago has failed to translate into approved anti-infective agents in the clinic. The only AMPs approved for medical use are gramicidin, nisin, daptomycin and its derivatives and polymyxins [99]. However, AMPs have been continuously searched, isolated, modified and/or incorporated in novel formulations aiming at high stability, low toxicity and high therapeutic index. This review will focus on novel formulations and assemblies for peptides and AMP.

## 2. Novel Formulations for Peptides and the Cyclosporin Case

**Figure 4:**
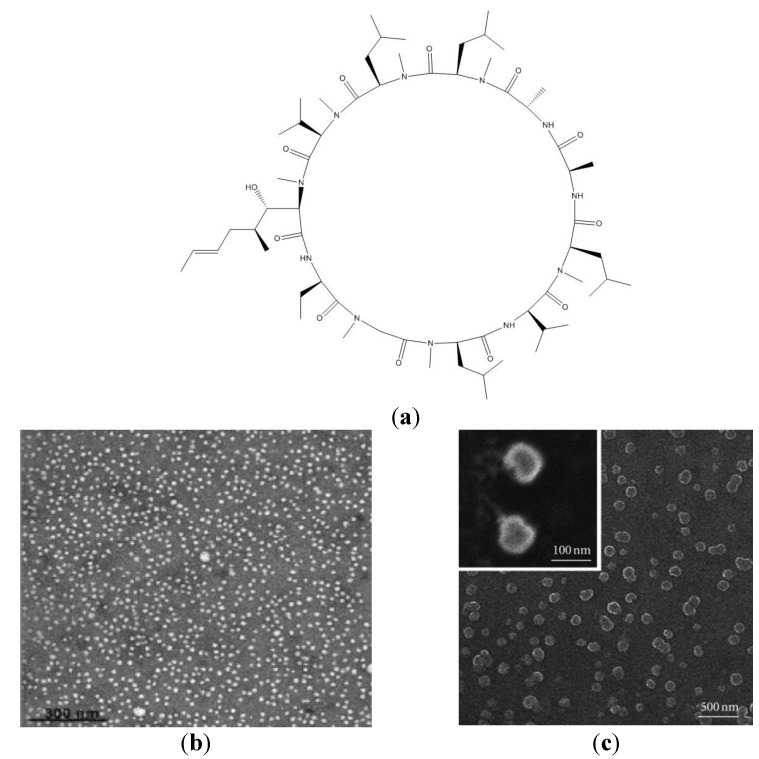
(**a**) The chemical structure of CsA. (**b**) Transmission electron micrographs of polymeric nanometric micelles carrying CsA. The bar corresponds to 300 nm. Adapted from [119] with permission from copyright 2011 Elsevier; (**c**) Scanning electron microscopy of polymeric nanoparticles of PEG-PLGA carrying CsA. Adapted from [120].

Cyclosporin A (CsA), a cyclic undecapeptide with several hydrophobic amino acid residues and immunosuppressive activity, is a good example of an extensively formulated peptide with more than 5000 publications reporting clinical trials and a large variety of formulations. A survey on novel cyclosporine formulations might provide meaningful examples of novel peptide formulations improving CsA therapeutic index and bioavailability. CsA chemical structure taken from [100] is on Figure 4a.

CsA from *Tolypocladium inflatum* is non-ribossomally synthesized by this fungus and widely used in the treatment of arthritis, psoriasis and as an immunosupressor preventing rejection of transplanted organs [101]. CsA antagonizes the activity of calcineurin, a calcium-dependent serine-threonine phosphatase, which dephosphorylates and activates a transcription factor (NFAT) that stimulates interleukin-2 (IL2) expression. Therefore, the dephosphorylation of NFAT is inhibited and consequently the IL2-dependent T cell proliferation is repressed. The therapeutic potential of CsA is limited mainly during long-term treatment due to several side effects, such as hepatotoxicity, nephrotoxicity, neurotoxicity and cytotoxicity [102]. Novel formulations leading to a specific mode of action can reduce toxicity and improve stability, bioavailability and efficacy *in vivo*. Lipids and surfactants can be very useful to formulate lipophilic substances in general and easily interact with the hydrophobic moieties of CsA or AMP. Particles are popular vehicles for CsA (and AMP, eventually) with more than 150 references on such CsA formulations and excellent reviews are available [103,104,105,106,107,108,109,110,111,112,113,114,115,116]. Lipidic or polymeric systems combine well with CsA. By spraying and drying, CsA alone yields large crystals whereas this same procedure applied to CsA in the presence of phospholipids yields much smaller and homogeneous microspheres of the peptide stabilized by the phospholipids [117]. CsA in lipids further surrounded by a pegylated-chitosan layer also produces small and well-dispersed particles in water solution [118]. The finest CsA dispersion in water solution are the nanometric, polymeric micelles of methoxy poly (ethylene glycol)-hexylsubstituted poly (lactide) (MPEG-hexPLA) yielding very small nanoparticles [119].

CsA in poly (ethylene glycol)-b-poly(d,l-lactide-co-glycolide) (PEG-PLGA) nanoparticles (NPs) smaller than 100 nm in diameter with good colloidal stability in salt solution were recently obtained by nanoprecipitation of CsA and PEG-PLGA [120]. These NPs stabilized by bovine serum albumin for the convenience of storage and transportation released 55.6% of CsA on day 1 after injection and showed potential for maintaining therapeutic CsA concentrations* in vivo*. The novel formulation efficiently suppressed T-cell proliferation and production of inflammatory cytokines dose dependently [120]. Accordingly, CsA-loaded poly (lactic acid)-PEG micro- and nanoparticles also released the peptide by a diffusion-mediated mechanism [121]. Figure 4b,c show the nanometric polymeric micelles [119] and NPs used to formulate CsA [120], respectively.

The methoxy poly (ethylene glycol)-hexylsubstituted poly (lactides) (MPEG-hexPLA) micelle formulations [106] shown in Figure 4b) were also studied* in vitro* against human corneal epithelial cells and in rabbits for the delivery of CsA to the eye [119]. Apoptosis was absent for the cells treated with the loaded or unloaded micellar dispersions. The observed corneal surface submitted to the formulations was comparable to the control surface treated with a 0.9% NaCl solution only. These MPEG-hexPLA/CsA NPs are promising formulations for ocular diseases such as dry eye syndrome and autoimmune uveitis, or for the prevention of corneal graft rejection [119]. In another study, an established rat model for the prevention of cornea graft rejection after a keratoplasty procedure was used [122]. After instillation of the MPEG-hexPLA/CsA formulation with fluorophore-labeled micelles, confocal analysis of flat-mounted corneas clearly showed that the nanosized carriers were able to penetrate into all corneal layers [122]. The efficacy of a 0.5% CsA micelle formulation was tested and compared to a physiological saline solution and to a systemic administration of CsA. The topical treatment for 14 days led to a significantly higher cornea transparency and lower edema after 7 and 13 days of the surgery compared to the control group. The success rate of cornea graft transplantation was 73% in treated animals against 25% for the control group. This result was similar to the one obtained for CsA parenteral administration in the same animal model but without the serious CsA systemic side effects [122]. 

CsA is poorly soluble in water. The first CsA formulations were oily, yielding low CsA bioavailability due to the preferential attraction of the drug to the lipophilic carrier rather than to the hydrophilic target tissue [123,124,125]. The first and only US FDA approved and commercially available formulation Restasis^®^ is a 0.05% CsA oil-in-water emulsion that does not deliver CsA to the corneal tissue [126]. Alternative formulations are the polymeric MPEG-hexPLA/CsA micelles [118,119,122], the cationic oil-in-water nanoemulsions leading to approximately 350 ng CsA /g cornea, 3 h after a single instillation [127,128,129] and the nonionic micelles of Solutol HS15 (poly oxyethylene esters of 12-hydroxystearic acid) with low toxicity *in vivo* [130]. Polymeric micelles [106,122] and cationic emulsions significantly increase CsA tissue levels after single and multiple dosing* in vivo* [127,128,129]. The nonionic micelles solubilize CsA avoiding the disadvantages of lipophilic solvents. Other marketed CsA formulations Sandimmune^®^ (Novartis International AG, Basel, Switzerland) and Neoral^®^ (Novartis International AG, Basel, Switzerland) containing surfactants and alcohol have limitations both in product safety for the parenteral, and in shelf life for the oral, administration [131].

Liposomal CsA has also been produced [132,133,134,135,136,137,138,139]. However, most of the currently available methods to generate liposomes are not very suitable for an industrial scaling-up and require the use of organic solvents [140]. Recently, a novel liposomal CsA was prepared using the supercritical fluid of carbon dioxide (SCF-CO_2_) to replace the organic solvent with the advantages of being nontoxic and inexpensive, yielding multilamellar phosphatidylcholine (PC) liposomes to carry CsA and opening the possibility of easy scaling-up [139].

Based on the improved solubility of CsA in ethanol, a novel CsA formulation in milk was described [141]. The high ethanol content in ethanol-water mixtures increased the solubility of cyclosporine so that a 100 mg cyclosporine-milk formulation administered orally to healthy volunteers showed satisfactory* in vivo* performance. The strong buffering capacity of milk and the high solubility of CsA in ethanol allowed the preparation of drug-milk formulations with enhanced pharmacokinetic properties [141].

Polymeric NPs, microspheres, hydrogels, and lipid-based delivery systems, such as liposomes, oil-in-water emulsions and solid lipid nanoparticles (SLNs), are all examples of carriers for CsA (and peptide) delivery.

## 3. Novel Formulations for AMP

Similarly to CsA formulations, the novel AMP formulations appearing in the nanotechnology domain also include lipids, liposomes, polymers, micelles, NPs, nanocapsules and other colloidal drug delivery systems of sizes up to a few hundred nanometers that have been loaded with AMPs and used as transporters to deliver AMPs to infected cells. Analogously to antibiotics [142], the AMP cargo has to reach the intracellular pathogens. Intracellular microorganisms are usually found in the phagocytic cells (neutrophils, monocytes), which are circulating in blood or inside the macrophages in the liver, spleen, lungs and other organs. Intracellular bacteria can invade also the central nervous system by using various mechanisms to overcome the blood-brain barrier (BBB), thus causing severe and often deadly sicknesses [143]. Any foreign microorganism that enters the blood circulation adsorbs various proteins from the blood plasma (albumin, antibodies, complement factors* etc.*), which may trigger the destruction of the microbe by making it recognizable by phagocytic cells able to engulf the pathogen. In the phagolysosome, the bacterium is submited to various killing mechanisms such as the production of reactive oxygen species and the action of lytic enzymes. However, some pathogenic bacteria can avoid recognition and phagocytosis whereas others can survive inside the phagocyte [144]. Some bacteria can even kill the phagocyte by releasing various toxic substances such as cytolysins, streptolysines* etc.*

Higher eukaryotes require the action of a complex network of cellular effectors of the immune system to recognize and eliminate the microbial invaders resident inside the cells [145]. Nanocarriers physically adsorb various proteins from the biological millieu and this adsorption determines the nanocarrier biodistribution and fate* in vivo* [146]. The interactions between nanocarrier and plasmatic proteins are fundamental intermolecular forces, such as van der Waals attraction, electrostatic interactions, hydrophobic interactions, hydrogen bonding and the short-ranged and repulsive hydration (solvation) forces [147]. Upon injection, the hydrophilic and non-charged carriers avoid the adsorption of serum proteins (albumin, immunoglobulins and complement factors), thereby preventing phagocytosis and exhibiting prolonged circulation in the blood stream as desirable for cancer chemotherapy [148,149]. In contrast, hydrophobic and positively charged carriers adsorb large amounts of negatively charged serum proteins [150]. This determines rapid recognition and phagocytosis, which concentrates the carriers and their cargo inside the macrophages of the reticuloendothelial system (liver, spleen, lung and other filtration organs) and the circulating phagocytic blood cells (monocytes and neutrophils). Thereby the desirable selective AMP delivery to the pathogens also inside the phagocytes occurs [151]. For instance, the AMP vancomycin administered systemically as such is unable to kill methicillin-resistant *S. aureus* (MRSA) inside macrophages [152] but becomes very effective when formulated in liposomes of 1,2-distearoyl-sn-glycero-3-phosphocholine (DSPC) and cholesterol [153]. PEGylated lipids in the liposomal formulation hamper AMP uptake by the macrophages thereby inhibiting AMP activity against intracellular MRSA [153].

The oral route of administration for peptides in general has to consider the proteolytic degradation by the enzymes of the gastro-intestinal (GI) tract. Oral formulations for peptides have to provide protection against degradation by enzymes. Both polymeric and lipidic carriers are able to encapsulate AMP thereby allowing its uptake by the enterocytes. After absorption, the carrier will slowly degrade according to a kinetic profile depending on its nature, thus providing a sustained and controlled release of the peptide [115]. Particles can cross the intestinal wall, although only in minute quantities [115]. The size and nature of the carrier nanoparticles are critical parameters involved in particle uptake by the GI tract. However, for many cyclic peptides such as CsA and other cyclic lipopeptides, resistance to proteases has been reported [97] and this might become important for novel oral formulations. Along similar lines, AMP modified with hydrophobic endings became resistant to proteases [154]. Overall, modification of proteins (and peptides) by hydrophobic fatty acids residues or amphiphilic block copolymers has been recognized as a promising and relatively safe strategy to deliver proteins (and peptides) to the brain [155]. On the other hand, similarly to enzymes [156,157,158,159], some loss of activity may occur upon peptide immobilization [160,161]. *C*-terminal immobilization distinctly reduced the activity of potent antimicrobial sequences though the correlation between the antimicrobial activity and structural properties such as amphipathicity and the structure-related activity profile of the investigated peptides did not change with immobilization [161].

The pulmonary membrane is naturally permeable to small molecules and many peptides and proteins [162,163]. The epithelium of the lung, the significant barrier to absorption of inhaled drugs, is thick (50–60 µm) in the trachea, but diminishes in thickness to an extremely thin 0.2 µm in the alveoli [163]. Some AMP formulations based on lipids or polymers can protect them from breakdown by peptidases in the lung and assure proper delivery. Anionic dimyristoyl phosphatidylcholine (DMPC) and dimyristoyl phosphatidylglycerol (DMPG) (3:1 molar ratio) liposomes encapsulated high levels of the cationic, α-helical CM3 AMP (730 microg/mL using 30 mM lipid concentration) and delivered CM3 by nebulization to the lungs of rats chronically infected with *P. aeruginosa* [164]. There was a reduction in the AMP toxicity and enhanced protection of the peptide against proteolytic degradation [164]. However, a similar liposomal formulation for polymyxin B sulfate using a combination of DMPG and non-ionic surfactants similar to PEG (Span 20 and Tween 80) presented reduced activity against *P. aeruginosa* strains [165]. NPs of <0.26 μm provide sustained drug release and are able to move to the alveolar epithelium without severe phagocytosis in alveoli or rapid elimination by the mucociliary clearance [166]. Recently, the potential of long-acting inhalable chitosan-coated PLGA nanoparticles containing hydrophobically modified exendin-4 for treating type 2 diabetes was disclosed [167]. Exendin-4 is a peptide similar to glucagon that promotes insulin gene transcription, synthesis and secretion, inhibits the apoptosis of pancreatic β-cells at the gene level, promoting their proliferation and regeneration, and reverses the development of diabetes [168]. Inhalable nanoparticles composed of glycol chitosan [169], chitosan-coated PLGA [170], or polybutylcyanoacrylate [171] also have sustained-release characteristics for calcitonin [169,170], insulin [171], and exendin-4 [167], and prolonged therapeutic effects in osteoporosis and diabetes [172]. Nanoparticle-mediated delivery of peptides and drugs to the lungs [104] and biomedical and pharmaceutical applications of polymeric AMP carriers have recently been reviewed [173].

The desirable characteristics for polymeric AMP carriers are biodegradability and bioresorbability. They should undergo hydrolysis to produce non-toxic compounds metabolized* in vivo* and in the environment [173]. AMP adsorb, self-assemble or can be covalently linked into a variety of polymeric materials such as natural polymers (e.g., alginic acid, chitosan, collagen, fibrin, gelatin, hyaluronic acid, polyhydroxyalkanoates, starch) and synthetic polyesters such as polylactide (PLA), polyglycolide (PGA), poly(ε-caprolactone) (PCL) and poly(γ-valerolactone) (PVL) or copolymers as PLGA [173]. Several polymeric materials are useful as carriers for a variety of AMP [173].

Nisin added directly to food loses its antimicrobial activity due to enzymatic degradation by food components [174]. Encapsulation technology protects this AMP and enhances its stability in foods [175,176]. Several formulations of nisin in liposomes [176,177,178,179,180], solid lipid nanoparticles [181], chitosan-carrageenan [182], or zein polysacharides nanocapsules were reported [183]. As a cationic AMP, nisin incorporation in negatively charged liposomes is favoured and anionic vesicles with dicetylphosphate in their composition yield the highest nisin encapsulation efficiency (50.1%–54.2%) whereas cationic vesicles with stearylamine in their composition show lower nisin encapsulation efficiency (11.7%–13.6%) [184]. Furthermore, the liposome charge also influences the interaction between liposomal AMP and bacterial cells, since bacterial cells are negatively charged and would repel similarly charged vesicles and attract oppositely charged ones [185]. Nisin in liposomes of soybean PC was less efficient against* L. monocytogenes* than free nisin [186]. Since unpurified soybean PC contains negatively charged fatty acids, partial purification of this lipid mixture has recently shown to improve the release of nisin from the liposomes and its efficacy against *L. monocytogenes* [187]. Nisin encapsulated in partially purified soybean PC (PC-1) or PC-1-cholesterol (7:3) liposomes presented, respectively, 218 and 158 nm diameters, zeta potential of −64 and −53 mV, and entrapment efficiency of 88.9% and 100%, due to their high zeta-potentials. All treatments reduced the population of *L. monocytogenes* compared to the control during 21 days of cheese storage at 7 °C. However, nisin encapsulated in PC-1-cholesterol liposomes was less efficient in controlling *L. monocytogenes* growth in comparison with free and PC-1 liposome-encapsulated bacteriocins [187]. The highest inhibitory effect occurs for nisin encapsulated in PC-1 liposomes after 10 days of storage of the product. The encapsulation of bacteriocins in liposomes of partially purified soybean PC may be a promising technology for the control of foodborne pathogens in cheeses [187]. Nisin and other bacteriocins are not active against Gram-negative bacteria due to the lipopolysaccharidic (LPS) barrier preventing AMP interaction with the cytoplasmatic membrane. However, chelating agents, such as ethylene diaminotetraacetic acid (EDTA), withdraw the divalent magnesium and calcium ions of the LPS, causing destabilization of the LPS layer, favouring the AMP interaction with the bacterium membrane and broadening AMP spectrum of activity [188,189]. Other approaches to enhance the activity of antibiotics or AMPs carried by liposomes have been the use of Ca^2+^ ions and/or fusogenic lipids such as 1,2-dioleoyl-sn-glycero-3-phosphatidylethanolamine (DOPE), which increase fusion between liposomal AMPs and the bacterium outer membrane [190]. Vancomycin in fusogenic liposomes was successfully delivered to the periplasmic space of Gram-negative bacteria thereby showing an antibacterial activity that was absent for the free AMP or for the non-fusogenic liposomes carrying the AMP [191]. Other liposomal formulations for vancomycin with PEGylated [192] or non-PEGylated phospholipids carrying vancomycin improved the AMP efficacy against MRSA in a rat model of infection [193]. Liposomal AMPs indeed improve AMP killing of bacteria engulfed by phagocytic cells also augmenting the elimination of macrophage-engulfed MRSA [153,194] but eventually not killing extracellular *S. aureus* [194]. In a particular formulation, porous nano-hydroxyapatite/chitosan/konjac glucomannan (*n*-HA/CS/KGM) scaffolds were loaded with cationic liposomal vancomycin [195]. This complex formulation provided sustained release yielding a better inhibitory activity on the formation of *S. aureus* biofilms in comparison with scaffolds without loaded liposomal vancomycin and showing potential for treating osteomyelitis caused by biofilm infections [195].

Liposomal formulations have been very sucessful and appraised in recent reviews on AMP delivery since artificial phospholipid vesicles are biocompatible, biodegradable, and nontoxic and able to entrap and carry hydrophilic, hydrophobic, and amphiphilic molecules to their site of action [196]. Liposomal polymyxin B formulations are also effective against multidrug-resistant (MDR) Gram-negative bacteria [197]. This AMP was incorporated into sonicated liposomes composed of either 1,2-dipalmitoyl-sn-glycero-3-phosphocholine (DPPC) and cholesterol (Chol) or 1-palmitoyl-2-oleoyl-sn-glycero-3-phosphocholine (POPC) and Chol with entrapment efficiencies of (32.1% ± 2.43%) and (5.35% ± 0.32%). The antimicrobial activity of the AMP in the sonicated DPPC/Chol liposomes against Gram-negative strains was generally higher than the free AMP [197]. Immunocytochemistry and electron transmission microscopy revealed that the penetration of polymyxin B into a resistant strain of *P. aeruginosa* is higher following its administration as a liposomal formulation as compared to its conventional form [197]. In an animal model of pulmonary infection, treatment with polymyxin B incorporated into liposomes composed of DPPC and Chol (2:1) significantly reduced the pulmonary bacterial counts as compared with that of free polymyxin B [198]. The levels of polymyxin B in the lungs of the infected animals treated with the liposomal dispersion were significantly higher (42.8 ± 6.2 microg/paired lungs) compared with those treated with the free drug (8.2 ± 0.4 microg/paired lungs) [198]. The direct delivery of liposomal polymyxin B to the lung effectively treated the pulmonary infection with *P. aeruginosa* by enhancing retention of the antibiotic in the lung. As a polycation, polymyxin B displaces magnesium and calcium ions from the outer LPS layer of Gram-negative bacteria and binds to LPS. However, in cystic fibrosis (CF), the CF sputum contains excreted polyanionic bacterial endotoxins and glycoproteins from lysed white blood cells, which have high affinity towards cationic AMP such as polymyxin B. These polyanions interact strongly with the free polycationic AMP inhibiting the AMP activity against the bacteria in the lungs. Polymyxin B entrapment in 1,2-Dimyristoyl-sn-Glycero-3-Phosphocholine (DMGPC) or DPPC and Chol reduced antibiotic inhibition up to 100-fold and the colony forming unities (CFU) counts of endogenous *P. aeruginosa* in sputum by 4-fold compared to the free AMP, suggesting their potential applications in CF lung infections [199]. In a murine pneumonia model, neutropenic mice infected with a clinical MDR *P. aeruginosa* strain and treated by intravenous administration of liposomal AMP had a significantly lower bacterial burden and a prolonged survival as compared to the control group receiving the free AMP also due to improved AMP penetration in the bacterial cells [200].

Bilayer disks are a much less used class of lipid-based nanocarriers for AMP [201,202]. They are prepared by sonication of charged lipids or by the disruptive effect of PEGylated lipids on liposomes [202] and have been employed to incorporate gramicidin A [27,28] or mellitin [203,204,205]. The cationic bilayer disks of the synthetic lipid dioctadecyldimethylammonium bromide (DODAB) [28] or of the lipid composition DODAB/DPPC [27] quantitatively incorporated gramicidin A and broadened the antimicrobial spectrum of this AMP [28]. The DODAB synthetic lipid by itself has high microbicidal activity against Gram-negative bacteria being less effective against the Gram-positive ones [206,207,208] or against fungi [209,210,211,212]. On the other hand, gramicidin A (Gr) is a poorly water soluble AMP that displays high toxicity against eukaryotic and mammalian cells and no activity against Gram-negative bacteria [29]. The DODAB/Gr combination conveniently displayed microbicidal activity against both Gram-positive and Gram-negative bacteria* in vitro* [28] and these formulations still require further testing* in vivo*.

**Figure 5:**
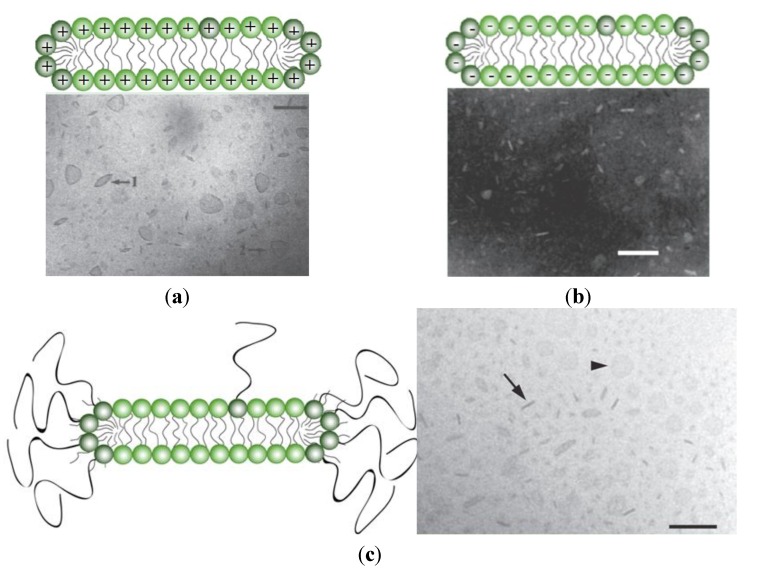
(**a**) Cationic bilayer disks of DODAB from cryo-transmission electron microscopy (cryo-TEM). The bar corresponds to 100 nm. Adapted from [213] with permission from 1995 American Chemical Society; (**b**) Anionic bilayer disks of sodium dihexadecylphosphate (DHP) from negatively stained dispersions seen by TEM. The bar corresponds to 100 nm. Adapted from [214] with permission from 1991 American Chemical Society; (**c**) Neutral phospholipid disks of POPC/Chol/ceramide-PEG5000 (35:40:25 mol %) from cryo-TEM. The bar corresponds to 100 nm. The arrow and arrowhead indicate disks observed edge-on and face-on, respectively. The bar corresponds to 100 nm. Reprinted from [203] with permission from Elsevier, Copyright 2011.

PEG-stabilized lipid disks can incorporate the AMP melittin [203]. The AMP retains its ability to redistribute from the disk into a new host membrane and the formulation does not affect melittin’s capacity to induce membrane permeabilization. Further, the peptide incorporation in the disks provides fully protection against tryptic degradation [203]. Time-kill experiments revealed that all the antibacterial effects of melittin administered in free form was gone after a single exposure to *E. coli*. In contrast, the disk formulation showed significant bactericidal effect also upon a second exposure to bacteria, indicating an extended release of peptide from the lipid disks [203]. Other linear α-helical antimicrobial peptides such as magainin 2 and alamethicin also displayed high affinity for the PEG-stabilized lipid disks [204,215]. Of note, the peptides bind more strongly to disks than to liposomes produced from the same lipid components and the maximum loading capacity of the disks was superior to that of liposomes. In the case of melittin, the maximum peptide to lipid ratio was about ten times higher in disks than in the corresponding liposomes [204]. The higher binding of the peptides to the disks compared to the liposomes is understandable from the fact that the total surface area available for the bilayer/peptide interaction is doubled using the disks instead of the closed liposomal bilayers. Further, alamethicin and magainin, similarly to melittin, have a very high affinity for curved lipid surfaces [215]. Melittin induces lipid structures with high positive curvature [216,217,218]. Figure 5 shows schematic representations and images of cationic, anionic and neutral bilayer disks where the disks are either face-on or edge-on.

Among the polymeric systems, the polymersomes mimic the liposomes as carriers for peptides and proteins to the brain [219]. They are capsules prepared from copolymers such as the biodegradable PEG-PLGA that can adsorb the lactoferrin peptide and cross the BBB to deliver the peptide to the central nervous system since lactoferrin specifically binds to several receptors in the brain tissue [219].

As applications in the dentistry field, some polymeric particles and micelles are able to bind to minerals such as those on the teeth surface and deliver encapsulated AMP over a prolonged period of time [220,221]. These novel formulations prevent the formation of *Streptococcus mutans* biofilm on the tooth surface and reduce the viability of the preformed biofilm even in the presence of a saliva pellicle layer [222]. Because of their excellent biocompatibility and biodegradability, diphosphoserine peptide and pyrophosphate were the tooth-binding moieties and these micelles were prepared by self-assembly of the peptides and the antimicrobial agent triclosan. The drug deliverance with a sustained release profile suggests that saliva proteins have minimal influence on triclosan release from the micelles. However, the influence of saliva, when used clinically, is essential for the binding of micelles to the tooth surfaces, and success of the treatment, since both diphosphoserine and pyrophosphate binding moieties compete with salivary proteins for binding sites on the tooth surface. Hence, the optimal application period of this peptidic micelle formulation should be immediately after tooth brushing [220]. Furthermore, this kind of micelle could also carry AMP as the active agent to act as an antiplaque formulation. 

A small and angiogenic α-helical, cationic peptide AG-30, with both antimicrobial and pro-inflammatory activities, was developed and formulated into a slow release system using biodegradable freeze-dried cationic gelatin microspheres, yielding a formulation with potential for treating ischaemic diseases [223]. With an anionic gelatin the AG-30 peptide was released slowly. This novel peptide induced angiogenesis and had antimicrobial activity inducing the lysis of bacterial cells without affecting eukaryotic cells [224]. This selectivity of AG-30 allows the concomitant killing of bacteria and enhancement of endothelial cell growth [223]. As the free AG-30 peptide is unstable and easily degraded by proteases* in vivo*, the slow-release formulation in gelatin microspheres was effective in protecting the peptide, increasing its stability and allowing the extended delivery of the peptide in a mouse ischaemic hind limb model, for angiogenic and antimicrobial treatment. These AMP-gelatin microspheres have also enabled the controlled release of AG-30 in muscle over a period of 2 weeks in response to a single injection of the formulation: the release was due to the enzymatic degradation of the gelatin microspheres [223]. Gelatin, used as an AMP carrier, possesses variable charge (by altering the processing method of collagen) [225] allowing modulation of degradation rates and/or the interactions between the AMP and the gelatin molecules [226].

**Figure 6:**
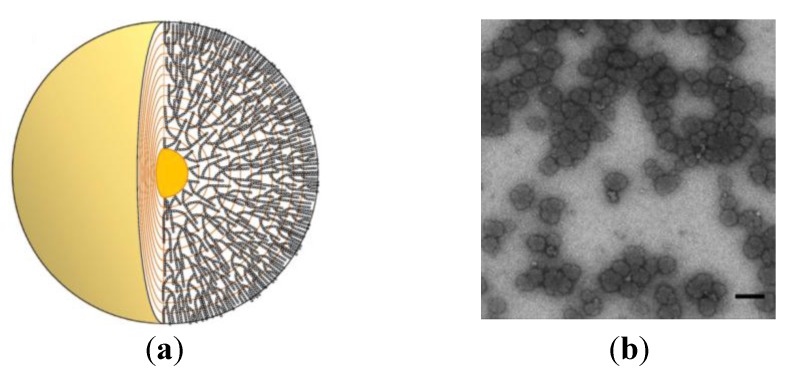
(**a**) Schematic illustration of a phytoglycogen (PGG) nanoparticle; (**b**) TEM images of the PGG dispersion. The scale bar corresponds to 100 nm. Adapted from [227] with permission from 2011 Elsevier.

Phytoglycogen (PGG) nanoparticles can carry nisin [227]. PGG is a water-soluble glycogen-like α-d-glucan from plants [228,229]. These novel nisin nanocarriers were prepared from PGG polyssacharide nanoparticles subjected to β-amylolysis and subsequent succinate or octenyl succinate substitution, combined or not with β-dextrin (PGB) [227]. The succinate substitution brings negative charges, and octenyl succinate substitution brings negative charges and hydrophobicity to the nanoparticles [230]. The properties of PGG derivatives depend on the degree of substitution. PGB-based nanoparticles showed a greater capability to retain nisin activity than did PGG-based ones, regardless of the substitution with succinate or octenyl succinate. The surface thinning of nanoparticles due to β-amylolysis resulted in increased nisin loading, leading to prolonged activity of the formulation against *L. monocytogenes*. The degree of substitution, hydrophobicity, and glucan structure affect nisin loading and release [227]. PGG-based nanoparticles from TEM are shown in Figure 6.

A novel class of nanoparticles was developed from the self-assembly of an amphiphilic peptide, showing a broad spectrum of high antimicrobial activity against a range of bacteria, yeasts and fungi [231]. This peptide can easily form core-shell structured nanoparticles (micelles), having a hydrophobic cholesterol core, to better drive self-assembly and improve membrane permeability of cholesterol-incorporated materials [232] and a hydrophilic cationic peptide shell containing cell penetrating peptidic sequence and arginine residues for adding cationic charges and improving membrane translocation [233]. These nanoparticles yield a high therapeutic index against *S. aureus* infection in mice, displaying more potency than the isolated peptide and being able to cross the BBB to suppress bacterial growth in the brain [231]. In fact, some AMPs are active against pathogens such as the yeast *Cryptococcus neoformans* responsible for a form of meningitis [234]. The treatment in these cases is complicated, since there is a poor penetration of most drugs across the BBB. The BBB is a layer of tight endothelial cells in the brain capillaries that limit the entrance of many molecules in the central nervous system (CNS). Surface-modified polymeric nanoparticles able to cross the BBB can deliver drugs that act on the CNS [235,236,237]. The enhancement of drug transport through the BBB from the coated nanoparticles takes place due to the binding of the nanoparticles to the endothelium of the brain capillaries followed by drug passive diffusion and/or nanoparticle phagocytosis [238].

Several cell-penetrating peptides (CPP) can act as targeting agents for nanoparticle functionalization due to their ability to translocate across cellular membranes via a mechanism independent of transporters or receptor-mediated endocytosis [239]. CPP are, in general, cationic or amphipathic sequences of, typically, up to 30 amino acids [240]. Interestingly, cationic CPP contain clusters of arginine and lysine residues, which make them very similar to AMP, suggesting that peptidic nanoparticles could be synthesized having both activities, antimicrobial and able to penetrate into cells [241]. The CPP deliver also cell-impermeable compounds into living cells and translocate various bulky cargos such as other peptides, proteins, siRNA, DNA, and nanoparticles across cellular plasma membranes [242,243].

Hydrogels can deliver small molecules such as antibiotics or be made of an antibacterial agent, circumventing the need to encapsulate therapeutics [244,245,246]. Antimicrobial hydrogels are also important in wound healing [247]. When infection prevents tissue regeneration at the site of injury, biocompatible hydrogels carrying AMP accelerate the healing by allowing cells attachment and infiltration [248,249,250]. Hydrogels are three-dimensional networks of ionic or neutral hydrophilic polymers physically and/or chemically crosslinked and able to swell by imbibing water [251,252]. They can respond to variations in pH, ionic strength or temperature with dramatic changes in volume, network structure, permeability, or mechanical strength. This inspired the design of several biocompatible drug delivery systems [251,252,253,254] releasing the encapsulated drug upon swelling of the hydrogel [255,256,257,258]. The synthetic peptide PXL150 with broad-spectrum antimicrobial activity incorporates well into a hydroxypropyl celulose gel for topical treatment of infected wounds at the surgical sites [259]. PXL150 is a novel short synthetic AMP, active against Gram-positive and Gram-negative strains, including MRSA [260]. Hydroxypropyl celulose is a nonionic water-soluble polymer commonly used in pharmaceutics as a thickening agent [261]. *In vivo* the hydrogel allowed PXL150 slow release on the wound site [259].

**Figure 7:**
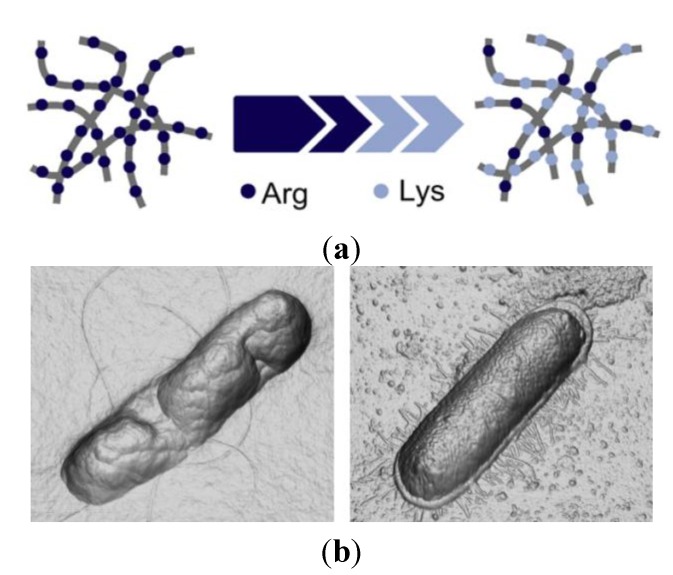
(**a**) Scheme of the fibril network of hydrogels with arginine gradual replacement by lysine; (**b**) The three-dimensional orthogonal projection images (derived from atomic force microscopy height data) of *E. coli* cells after 2 h interaction with PEP6R hydrogel surface (**left** image) or control surface (**right** image). Adapted with permission from [270], copyright 2012 Elsevier.

Antiseptic wound dressings often fail for chronic infections involving biofilms or resistant bacteria [262]. A gel formulation combined the antibiofilm enzyme Dispersin B^®^, the broad-spectrum AMP KSL-W and the gelling agent Pluronic F-127 [263]. Dispersin B^®^ is an enzyme produced by the oral bacterium *Aggregatibacter actinomycetemcomitans* [264] that not only inhibits biofilm formation but also disperses preformed biofilm [265]. The KSL-W is a cationic antimicrobial decapeptide [266,267] with antiplaque activity [268]. Pluronic F-127 is an innert block copolymer of poly (propylene oxide) and (ethylene oxide) that forms a semisolid gel at room temperature and a more fluid one at lower temperatures [269]. This enzyme-peptide wound gel reduced by 50% the minimal inhibitory (MIC) and bactericidal concentrations (MBC) against MRSA, *S. epidermidis* and *Acinetobacter baumannii* when compared to the activity of the free peptide. The sustained release of the peptide obtained with Dispersin B^®^-KSL-W peptide-based gel did not occur for the commercial wound gel Silver-Sept™. Dispersin B^®^-KSL-W peptide-based wound gel is effective in inhibiting the biofilm-embedded bacteria, thus showing potential clinical applications in wound and skin care products [263]. Other hydrogels have also been successful for formulating AMPs yielding bactericidal assemblies against Gram-positive and -negative bacteria plus MDR *P. aeruginosa* [270,271,272]. The fibril structure comprises a bilayer of hairpins linked by hydrogen bridges along the long-axis of a given fibril so that the solvent—exposed fibril surfaces display a high concentration of arginine side chains [270]. Self-assembling AMP for the construction of new materials usually allows an easy determination of the structure-activity relationships, since changes in the peptide sequence at the monomer level correlate with changes of the bulk material’s antibacterial properties. Antibacterial hydrogels were prepared using self-assembling AMPs with a high content of lysine. These lysine-rich AMPs assemble into polycationic fibrillar networks showing bactericidal properties via a mechanism involving bacterial membrane disruption. When the bacteria contact the fibril surface, their membranes undergo lysis [271,272]. In fact, materials with polycationic surfaces are effective against Gram-positive and Gram-negative bacteria, killing the bacteria upon contact by membrane disruption [245,273,274,275,276,277,278]. A special feature of these fibrillar materials is that their surface chemistry can be varied by changing the amino acid composition of the peptide monomer used for the self-assembly [271,272]. Thus, modifications on the structure of these gels at the nanometer length scale are effective to create new materials with enhanced activity. The polycationic surface of many AMP with high content of arginine residues drives the interaction with the anionic membrane surface of bacteria and bacterial cell lysis [279,280,281,282,283]. The effect of the arginine content on the antibacterial, hemolytic and rigidity of the gel was evaluated for a family of hydrogels based on the PEP8R peptide [270]. The PEP8R parent molecule is an amphiphilic β-hairpin peptide of twenty residues (eight of which are arginines, 8R) with side chains displayed on its hydrophilic face. This peptide self-assembles into a network of fibrils forming a moderately rigid hydrogel with potent activity against *E. coli*,* S. aureus* and MDR *P. aeruginosa* but also against human erythrocytes causing their lysis. This lack of selectivity led to the replacement of some arginines by lysines. The derivative AMPs with only four (4R) to six arginine residues (6R) displayed good antibacterial activity and low toxicity against the erythrocytes suggesting that the large number of arginines side chains is responsible for the hemolytic activity of the gel [270]. Furthermore, reducing the arginine content on the AMPs led to a decrease in the rigidity of the hydrogel [270]. The hydrogels obtained by gradual replacement of arginines by lysines and their effects on *E. coli* cells are illustrated on Figure 7. 

Peptide self-assembled systems, in which non-covalent interactions are responsible for the physical assembly of peptide molecules, offer a good and viable alternative to generate hydrogels [284]. However, the majority of peptides capable of self-assembly into hydrogels consist of rather long molecules (>10 amino acids) [285], for which solid-phase synthetic preparation is expensive and difficult to scale-up. In contrast, ultrashort peptides (*i.e.*, made of 2 or 3 amino acids) are attractive candidates for hydrogels, as they can be readily prepared in solution-phase, making scaling-up convenient [286]. Under physiological conditions, some ultrashort peptides [287,288] self-assembled as hydrogels in the presence of the antibiotic ciprofloxacin, a model of hydrophobic drug [289]. Ciprofloxacin contributed to the gel nanostructure yielding softer gels with increased stability as compared to the gels in absence of the antibiotic, displaying good activity against *S. aureus*, *E. coli* and *Klebsiella pneumoniae*, and infiltration of fibroblasts into the gel as desirable for the design of wound dressings [289]. In the absence of ciprofloxacin the peptide gel revealed a mild antimicrobial activity against the Gram-negative bacteria and no major effect against human erythrocytes or mouse fibroblast cells [289].

Chewing gums can carry antiplaque agents releasable into the saliva [290,291]. The dental plaque is a result of the interactions between teeth and adsorbed host or bacterial molecules with co-adhesion and multiplication of associated microorganisms [292,293]. AMPs were also incorporated in chewing gums as antiplaque formulations [294,295]. The cationic antimicrobial decapeptide KSL with five lysine residues has a broad-spectrum of antibacterial activity and inhibits the growth of oral bacterial strains associated with caries development and plaque formation [296]. The KSL-chewing gum formulations showed favorable* in vitro*/*in vivo* release profiles for the peptide yielding about 80% of peptide release in 20 min [294], the usual chewing time for more than 80% of the gum chewers tested in a U.S. study [297]. Though KSL is stable in artificial saliva and binds with high affinity on teeth-mimetic hydroxyapatite discs pretreated with artificial saliva [294], it is degradable by human saliva and by simulated gastric fluids. In order to improve stability against enzymatic degradation, the KSL-W derivative with the l-tryptophan, replacing the l-Lys6 residue of the KSL was introduced [267]. KSL-W resists the salivary trypsin in the oral cavity but is still degradable by the gastric and pancreatic enzymes assuring the safety in the gastrointestinal tract and the degradation before systemic absorption [267]. The chewing gum impregnated with KSL-W has excellent* in vitro* and* in vivo* releasing profiles, reaching up to 90% of sustained release within 30 min of chewing* in vivo* [295]. KSL-W remains stable for one hour in human saliva, has a strong affinity for human saliva-coated and uncoated hydroxyapatite disks and is degradable by gastric and pancreatic enzymes. Furthermore, the inclusion of the established antiplaque and antibacterial agent cetylpyridinium chloride in the KSL-W/chewing gum formulation further increases the rate of AMP release from the formulation [295].

The progress of septic shock in the clinic is prevented by cartridges immobilizing polymyxin B on polystyrene fibers for removal of bacterial endotoxin from the circulation [298,299,300,301]. Sepsis is a complex systemic inflammatory response to microbial pathogens. The presence of microorganisms in the bloodstream causes an innate immune response characterized by the stimulation of monocytes, release of proinflammatory cytokines and activation of different immune effectors [302]. The inflammatory response to control the infection causes local vasodilatation, release of various cytotoxic substances, and, eventually, destruction of the invading pathogen. Nevertheless, many of the same components of inflammation that are beneficial in host defenses against infection can be deleterious, causing cell and tissue damage and hence multiple organ failure [300]. Bacterial-associated toxins such as the endotoxins from the Gram-negative and the lipotechoic acid of the Gram-positive bacteria are somes of these deleterious substances released into the blood [303,304,305]. Conventional therapy such as antibiotics and surgical procedures to remove the source of infection is crucial for treating sepsis, but cannot reverse the effects of the bacterial toxins already released into blood or of the endogenous mediators produced by the host in response to bacteria [300]. Removing endotoxins is effective to manage severe sepsis. Thus, devices to remove endotoxin or inflammatory cytokines from the circulation have been designed to reduce the morbidity and mortality associated with sepsis [304,306,307]. Polymyxin B binds endotoxin through hydrophobic and electrostatic interactions since its hydrophobic amino acids (Phe, Leu) interact by the hydrophobic effect with the lipid A fatty acid moieties of the endotoxin whereas its positively charged amino groups form ionic bonds with lipid A negatively charged phosphates [308]. The high affinity between the immobilized polymyxin B and the endotoxin is responsible for the extracorporeal removal of the endotoxin from the patient circulation into the cartridge and avoids the significant nephrotoxicity and neurotoxicity caused by intravenous polymyxin B [309,310,311]. Its immobilization on polystyrene fibers of a hemoperfusion column or cartridge allows the endotoxin removal without its toxic effects [304,306,312,313]. Polymyxin is covalently bound to the polystyrene fibers by a reaction between one of the amino groups of the diaminobutyric acid residues, leaving at least three to four charged amino groups for LPS binding [314]. Polymyxin B bound and immobilized to polystyrene fibers (PMX) has been very useful against sepsis* in vivo* to purify the blood of infected patients by hemoperfusion [301,314]. The direct hemoperfusion (DHP) with PMX (DHP-PMX) treats patients (with endotoxemia or suspected Gram-negative infection), who fulfill the conditions of Systemic Inflammatory Response Syndrome and present septic shock requiring vasoactive agents [314]. Several studies already demonstrated the efficient removal of endotoxin from the circulation with DHP-PMX as well as suppression of *S. aureus* lipoteichoic acid-induced TNF-α production [314,315,316,317]. The treatment with DHP-PMX improves hemodynamics and organ dysfunction and reduces mortality rate in patients with severe sepsis arising from intra-abdominal infections with Gram-negative bacteria [304].

Table 1 shows some novel formulations for AMPs. Pre-clinical studies with native AMPs have been limited, partly due to the difficulty in generating or obtaining adequate amount of peptides and partly because of the unavailability of the established animal models. Only a few formulations have already undergone* in vivo* pre-clinical or clinical trials. Some examples are the *n*-HA/CS/KGM scaffold loaded with liposomal vancomycin for treating osteomyelitis [318], the gelatin microspheres carrying the AMP AG-30 for angiogenic and antimicrobial purposes [223], the hydroxypropyl cellulose gel combined with the AMP PXL 150 for the treatment of wound infections [259] and the cartridges of immobilized polymyxin B for septic shock therapy with good results in pre-clinical [300] or clinical trials [299]. Some AMPs [319], host defense peptides (HDP), HDP-based [320] or AMP-based formulations [99] undergoing preclinical or clinical studies have been reported. 

Novel formulations can avoid AMP non-specific toxicity, susceptibility to proteolysis and poor bioavailability [320,321]. Although only few AMP or AMP formulations underwent clinical trials, the resources coming from nanotechnology open new avenues for bringing AMP and their formulations from the bench to the bedside.

**Table 1:** Some examples of AMP formulations.

Carrier	AMP	Spectrum of Activity	Indication	Ref.
Aerosol DMPC/DMPG liposomes	CM3	*P. aeruginosa*	Pneumonia, lung infections	[164]
PEG-PLGA polymersomes	Lactoferrin	Not specified	Meningitis, brain-related infections	[219]
Liposomes	Nisin	*Lactococcus lactis*	Cheese manufacture	[180]
Liposomes	Nisin	*L. monocytogenes*	Cheese ripening	[178,179]
Fusogenic liposomes	Vancomycin	Gram-negative bacteria	Related infections	[191]
*n*-HA/CS/KGM scaffold for liposomes	Vancomycin	*S. aureus*	Osteomyelitis	[195]
DMGPC:Chol; DPPC:Chol liposomes	Polymyxin B	*P. aeruginosa*	Cystic fibrosis	[199]
DODAB liposome or bilayer disk	Gramicidin	*E. coli*, *S. aureus*	Related infections	[28]
POPC/cholesterol/ceramide-PEG5000 bilayer disk	Mellitin	*E. coli*	Related infections	[203]
Gelatin microspheres	AG-30	*P. aeruginosa*, *E. coli* and* S. aureus*	Anti-ischaemia, angiogenic and antimicrobial	[223]
PGG nanoparticles	Nisin	*L. monocytogenes*	Food preservation	[227]
Hydroxypropyl cellulose gel	PXL150	Gram-positive and Gram-negative bacteria, MRSA	Wound surgical site infections	[259]
Hydrogel + enzyme Dispersin B^®^	KSL-W	MRSA, *S. epidermidis A. baumannii*	Chronic wound infections with associated biofilm	[263]
Injectable peptidic hydrogel	PEP8R or derived with balanced arginine residues	MDR *P. aeruginosa E. coli* and* S. aureus*	Wound infections	[270]
Peptidic hydrogel + Ciprofloxacin	Tripeptide of Leu-Phe-Phe	*S. aureus*,* E. coli* and* K. pneumoniae*	Wound infections	[289]
Chewing gum	KSL	Oral bacterial pathogens	Dental plaque and caries	[294]
Chewing gum	KSL-W	Oral bacterial pathogens	Dental plaque and caries	[295]
Polystyrene fibers	Polymyxin B	Endotoxin of Gram-negative bacteria	Sepsis and septic shock	[298,299,300]

## 4. Conclusions and Perspectives

AMPs certainly require covalent modifications and/or novel formulations to become less toxic, more bioavailable and useful in the biomedical field. They usually display unspecific toxicity to cells, derived from their interactions with any bilayer membrane; thus their devastating power requires modulation. However, creative formulations for AMPs are within the limits of nanotechnology and, previously unenvisaged uses, even outside the limits of antimicrobial chemotherapy, including cancer, diabetes, transplantation, anti-angiogenesis, cell penetration, and cell targeting *etc.*, are opening new frontiers for AMPs. Old AMPs in new formulations or in new applications may become very useful. For example, multidrug resistant strains may not resist the physical mechanism of membrane disruption by AMPs; the challenge will be to deal with their huge diversity of structure and function.
